# Ticks and spirochetes of the genus *Borrelia* in urban areas of Central-Western Poland

**DOI:** 10.1007/s10493-024-00932-5

**Published:** 2024-06-28

**Authors:** Olaf Ciebiera, Renata Grochowalska, Andżelina Łopińska, Piotr Zduniak, Tomasz Strzała, Leszek Jerzak

**Affiliations:** 1 Institute of Biological Sciences, University of Zielona Góra, Prof. Z. Szafrana 1, Zielona Góra, 65-516 Poland; 2 Department of Avian Biology and Ecology, Faculty of Biology, Adam Mickiewicz University, Uniwersytetu Poznańskiego 6, Poznań, 61-614 Poland; 3 Department of Genetics, Wroclaw University of Environmental and Life Sciences, Kożuchowska 7, Wrocław, 51-631 Poland

**Keywords:** *Borrelia burgdorferi* sensu lato complex, Habitat, Poland, Tick-borne disease, Urban areas

## Abstract

Due to the extensive use of green urban areas as recreation places, city residents are exposed to tick-borne pathogens. The objectives of our study were (i) to determine the occurrence of ticks in urban green areas, focussing on areas used by humans such as parks, schools and kindergartens, and urban forests, and (ii) to assess the prevalence of *Borrelia* infections in ticks in Zielona Góra, a medium-sized city in western Poland. A total of 161 ticks representing the two species *Ixodes ricinus* (34 males, 51 females, 30 nymphs) and *Dermacentor reticulatus* (20 males, 26 females) were collected from 29 of 72 (40.3%) study sites. In total, 26.1% of the ticks (85.7% of *I. ricinus* and 14.3% of *D. reticulatus*) yielded DNA of *Borrelia*. The difference in the infection rate between *I. ricinus* and *D. reticulatus* was significant. Among infected ticks, the most frequent spirochete species were *B. lusitaniae* (50.0%) and *B. afzelii* (26.2%), followed by *B. spielmanii* (9.5%), *B. valaisiana* (7.1%), *B. burgdorferi* sensu stricto, (4.8%) and *B. miyamotoi* (2.4%). No co-infections were found. We did not observe a correlation in the occurrence of *Borrelia* spirochetes in ticks found in individual study sites that differed in terms of habitat type and height of vegetation. Our findings demonstrate that the *Borrelia* transmission cycles are active within urban habitats, pointing the need for monitoring of tick-borne pathogens in public green areas. They could serve as guidelines for authorities for the proper management of urban green spaces in a way that may limit tick populations and the potential health risks posed by tick-borne pathogens.

## Introduction

The degree of urbanisation has increased significantly over the last century, doubling and even quadrupling in various cities in developing countries (Goldstein 1990). It is estimated that 56% of the human population lives in cities and by 2050 this could be as high as 70% (worldbank.org). The observed rapid urbanisation is a significant threat to biodiversity through the overall modification and fragmentation of ecosystems. Several studies on particular species groups of birds, mammals, and plants along the urbanisation gradient indicated that species richness generally decreases (e.g., Melles et al. 2003; Riem et al. 2012; McKinney 2008). Consequently, urbanisation leads to biotic simplification and homogenisation of ecosystems (McKinney 2002, 2006). Furthermore, rapid human population mobility and migration, and contacts of people and their pets with wild vertebrate species, may contribute to changing epidemiological and epizootiological conditions in urban areas (Uspensky 2008). There is a strong tendency to improve the quality of life by developing urban biodiversity (parks, gardens, boulevards, community gardens, ventilation corridors) in city centres (Rega-Brodsky et al. 2022; Benedetti et al. 2023). The development of any urban area entails dramatic changes in the biodiversity of flora and fauna. It also affects human and animal disease vectors and has a strong impact on the interaction of wildlife with pathogens. The presence of ixodid ticks (especially in recreational areas and urban parks) has been confirmed in multiple studies across Europe (e.g. Buczek et al. 2014; Rizzoli et al. 2014; Nelson et al. 2015; Kowalec et al. 2017; Mathews-Martin et al. 2020; Hansford et al. 2021; Kocoń et al. 2022). In central Europe, ticks were collected from urban areas as well. In an urban park in Ostrava (Czech Republic), 13,8% of collected *I. ricinus* ticks were infected by *B. burgdorferi* s.l. (Venclíková et al. 2014). In urban areas of the Ukrainean city of Kiev 696 *I. ricinus* and 69 *D. reticulatus* were collected (Didyk et al. 2017). Ticks were also present in an urban-type settlement (Weiner 2018). Similarly, 670 *I. ricinus* ticks were collected from urban and suburban sites in southeastern and northeastern Slovakia (Pangrácová et al. 2013). *Ixodes* ticks were found in recreation areas in the urban area of Hanover, but the largest number of tick species were found in the urban area of Berlin (12 tick species) (Hauck et al. 2020; Rubel et al. 2022).

The city of Zielona Góra was chosen as a model city, which in the early 21st century almost quadrupled its boundaries from 57 km^2^ to 217 km^2^. The progression of urbanisation may be important in tick distribution because of how urban greenery is maintained and access to hosts such as small mammals and birds.

Lyme borreliosis is a systemic infection disease found in various cities around the world (Corrain et al. 2012; Buczek et al. 2014; Nelson et al. 2015; Kowalec et al. 2017; Mathews-Martin et al. 2020; Hansford et al. 2021; Kocoń et al. 2022). Etiological factors responsible for this disease are Gram-negative spirochetes of the complex of *Borrelia burgdorferi* sensu lato which includes 23 species (Steinbrink et al. 2022). In northern Poland, 14 different species of the genus *Borrelia* have been detected in *I. ricinus* ticks, including *B. afzelii, B. garinii, B. burgdorferi* sensu stricto, *B. bavariensis, B. spielmanii, B. bissettiae, B. lusitaniae, B. valaisiana, B. finlandensis, B. lanei, B. californiensis, B. carolinensis, B. americana*, and *B. turcica* (Wodecka and Kolomiiets 2023). The first five species are considered human-pathogenic. Furthermore, *B. bissettiae, B. lusitaniae*, and *B. valaisiana* have been identified in humans, however, their pathogenicity remains still unclear (Strnad et al. 2017; Steinbrink et al. 2022). Lyme disease symptoms include erythema migrans (EM), meningitis, acrodermatitis chronica atrophicans, peripheral neuropathy, arthritis and borrelial lymphocytoma (Stanek and Strle 2008). Ticks of the *Ixodes* genus are the primary vectors for spirochetes of the *B. burgdorferi* complex. *I. ricinus* is a primary vector for this pathogen in Europe, although they can be transmitted by other tick species like *I. persulcatus*, *I. hexagonus*, *I. canisuga*, *I. kaiseri* (Nowak-Chmura 2013). Lyme borreliosis spirochetes were also detected in *Dermacentor* genus in Europe, for example in *D. reticulatus* (Nowak-Chmura 2013; Mierzejewska et al. 2015; Kubiak et al. 2024).

*B. miyamotoi*, an emerging tick-borne pathogen in the Northern Hemisphere, was found in only one tick during this study. In Poland, its presence was found in ticks collected from plants (Wodecka et al. 2010).

Ticks distribution in the natural environment and the risk level of contact with tick-borne pathogens are the subject of many studies worldwide. In Poland, *B. burgorferi* was detected in *I. ricinus* ticks for the first time in 1993 near Olsztyn (Wegner et al. 1993). Investigations concerning the presence of *B. burgdorferi* in ticks were conducted also in Cracow, Lublin, Bydgoszcz and Warsaw areas (Siuda et al. 2002; Biaduń 2008; Błażejewicz-Zawadzińska et al. 2011; Kasprzak et al. 2016; Zając et al. 2017; Kowalec et al. 2017). The study by Siuda et al. 2002 showed the occurrence of ticks in most of the examined areas within the city limits of Kraków (13 from 18 localities). Ticks (only *I. ricinus*) occurred more frequently on the outskirts of the city in forest fragments (20–32 specimens per one hour of collection of ticks by flagging) than in the urbanized area (1–7 specimens per one hour of flagging), significantly influenced by human activities. In this study, no ticks were reported from the cultivated city parks and gardens. According to Biaduń 2008; no ticks were found in any of Lublin’s inner-city parks. However, they were present in the peripheral parts of the city in seminatural habitats such as extensive meadows, pastures, and various forest habitat types. Ticks occurred with marked intensity in habitats defined as ‘optimal’ in terms of humidity, shading and undergrowth, with the highest intensity in fresh pine forests (average of 75 individuals/sample), mixed forests (average of 49/sample), and young forests (average of 47 individuals/sample). In a study by Błażejewicz Zawadzińska et al. 2011, ticks were found in parks in Bydgoszcz but only specimen of *I. ricinus*. In a 2016 study by Kasprzak et al. 2016 in the Bydgoszcz Parks area, 415 ticks of *I. ricinus* were detected. The prevalence of *Borrelia* spirochetes was 12.5%. The predominant species was *B. afzelii*, followed by *B. burgdorferi* and *B. garinii*. In a study by Zając et al. 2017 in the Lublin area, 634 ticks of the species *D. reticulatus* were detected. The prevalence of *Borrelia* spirochetes was at the level 1.6%. In a study by Kowalec et al. 2017; the mean tick population in urbanised areas of Warsaw ranged from 0.9 to 10.1 ticks/100m^2^. The prevalence of spirochetes in urban areas was 10.9%. Six species of *Borrelia* sp. were detected: *B. miyamotoi*, *B. afzelii*, *B. burgdorferi*, *B. garinii*, *B. lusitaniae*, *B. spielmanii*, *B. valaisiana*. The predominant species in Warsaw was *B. afzelii* (69.3%). Sixteen cases of co-infection were detected. The risk of infection with *Borreliella* spp. and/or *B. miyamotoi* in highly- and low-transformed (in comparison with Białowieża natural forest) areas was similar.

Studies investigating tick distribution in the urban area of Zielona Góra revealed that ticks use urban areas, parks, cemeteries and recreation grounds as potential foraging grounds (Czupryk and Ciebiera 2014). Urban dwellers, especially people using green areas as recreation grounds have a high risk of being infected with borreliosis. According to the National Institute of Public Health in Poland, in 2021, almost 12,494 cases of borreliosis were recorded (https://www.pzh.gov.pl/). Therefore, it is necessary to monitor tick distribution, density and prevalence of *B. burgdorferi* s.l. and other pathogens infecting local tick populations (Kilpatrick et al. 2017; Vu Hai et al. 2014).

Our study aimed to determine the tick fauna and spirochetes of *Borrelia* spp. in an urban area of Zielona Góra. We focused on urban parks, schools and kindergartens, squares, and urban forests, i.e. areas places with high human activity.

## Materials and methods

### Study area

**Fig. 1:**
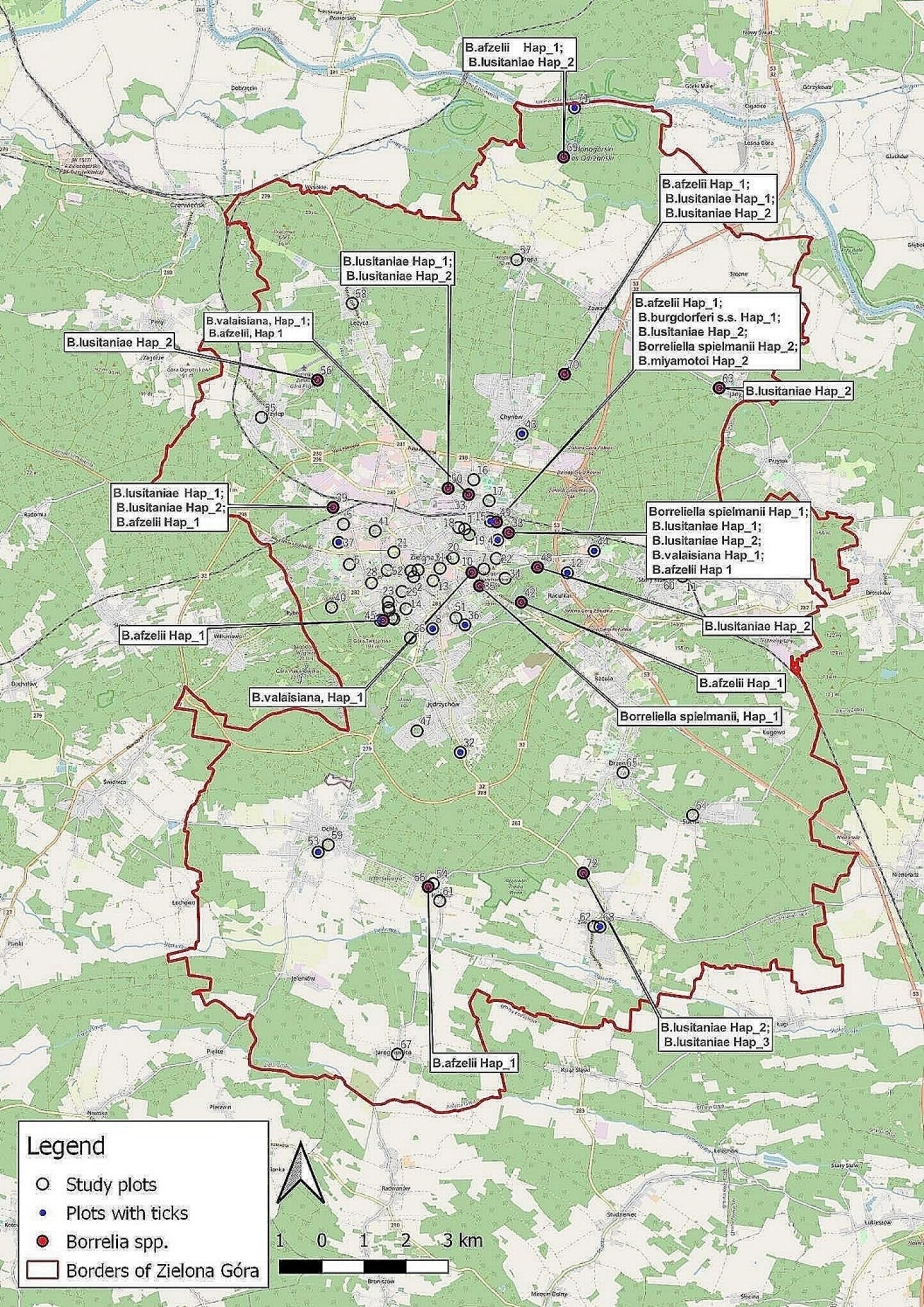
Locations of 72 study sites across Zielona Góra, W Poland along with information about Borrelia species identified in each study site (base map source: OpenStreetMap.org)

The study was carried out in Zielona Góra, a medium-sized city in western Poland (human population 140 708 (https://stat.gov.pl/) covering an area of 278.3 km^2^ (Fig. 1). A ring of communal forests surrounds the city. Due to the small number of water bodies, pine forest dominates here, and only in the area’s depressions are fragments of oak and ash-alder riparian forest. The total proportion of green areas in the city area is about 55%. The city is characterized by a strictly urbanized area of approximately 58.3 km^2^ (20%) and adjacent areas comprising forests, rural settlements and agricultural areas.

Within the administrative boundaries of the city, a total of 72 test plots of 100 m^2^ each (altogether 7,200 m^2^), were chosen and divided into four groups, depending on the degree of terrain transformation and dominant habitat type: (a) lawn areas within schools and kindergartens; (b) urban greenery: squares, flowerbeds, allotment gardens, cemeteries (characterized by a low density or even lack of trees and poorly developed undergrowth); (c) urban parks characterised by high shading of undergrowth and dominant deciduous tree species; (d) urban forests (the dominant type was dry pine forests, except two points where riparian forests on the Odra River occurred), which are recreational and leisure areas where city dwellers may be exposed to tick bites. All plots are located in key urban green spaces across the city and are regularly used by members of the public for recreational activities (walking with pets, sports activities, resting). Urban parks, greenery and forests provide refuges for more than 100 birds species (Indykiewicz et al. 2008) and mammals, including Red fox (*Vulpes vulpes*), Roe deer (*Capreolus capreolus*), Central European boar (*Sus scrofa*), European hedgehog (*Erinaceus europaeus*), Red squirrel (*Sciurus vulgaris*) and small rodents. Moreover, these areas are also used by dog owners for walking.

Ticks were collected twice in spring of 2021, from 17.03. to 20.04. and from 01.05. and 31.05. Ticks were sampled using the flagging method, i.e. the vegetation of each study plot was systematically and completely swept. All surveys were done only on dry days between 10 am and 3 pm and temperature above 7 °C. The average collecting time per study plot was about 15 min. During each survey, we recorded the vegetation height in the three categories low (up to 20 cm), medium (21–40 cm), and high (above 40 cm). Collected ticks were stored in Eppendorf containers with 70% ethanol for further study. Species identification of ticks followed the taxonomic keys of Siuda (1993) and Estrada-Peña et al. (2018).

### Nucleic acid extraction and PCR

The total DNA was extracted from each tick by lysis in ammonium hydroxide (NH_4_OH, Sigma Aldrich) (Rijpkema et al. 1996). The obtained lysates were kept at -20^o^C. In order to detect spirochetes of *Borrelia* spp. nested PCR was conducted. The protocol of Wodecka et al. (2010) was used to amplify 774- and 604-bp fragments of the *fla* gene, using primers 132f and 905r, and 220f and 823r, respectively. Amplification products were separated on 1% agarose gel stained with Midori Green DNA Stain (ABO, Poland). PCR-positive products were purified with the clean-up purification (EPPiC; A&A Biotechnology, Poland) and sequenced in both directions by using the same primer pairs (220f and 823r) by the firm SEQ.me (Czech Republic). All sequences were compared with the corresponding reference sequences deposited in GenBank using the NUCLEOTIDE BLAST NCBI program (www.ncbi.nlm.nih.gov.blast*).* Aligned sequences representing 42 *flaB* genes of *Borrelia* strains were examined with MEGAX software (version X; Kumar et al. 2018). To avoid contamination DNA isolation, the reaction mixture preparation and electrophoresis were carried out in separate rooms. A total of 42 sequences of the *flaB* gene were deposited in GenBank. The *flaB* gene sequences are listed as follows: OP776203-OP776213 (*B. afzelii*), OP776214-OP776215 (*B. burgdorferi* s.s.), OP776216-OP776236 (*B. lusitaniae*), OP776237-OP776239 (*B. valaisiana*), OP776240-OP776243 (*B. spielmanii*), OP776244 (*B. miyamotoi*).

### Statistical analysis

We analysed the influence of several factors possibly affecting the occurrence of ticks in the studied area: species [SPECIES], habitat type [HABITAT], vegetation height [VEGETATION] and the interaction effect of SPECIES and HABITAT using the Generalized Linear Mixed Model (GLMM) with Poisson distribution and log link function, where the number of ticks was the dependent variable and SPECIES, HABITAT, VEGETATION and SPECIES*HABITAT were the fixed factors, and the sampled plot [ID] and the control time of the plot [TIME] were random factors. Furthermore, we analysed the probability of occurrence [POBABLILITY] of Borrelia spp. in ticks recorded in the study sites concerning SPECIES, HABITAT, VEGETATION and SPECIES*HABITAT effects using Generalized Linear Model (GLM) with binomial distribution and logit link function, where PROBABILITY (binary variable) was the dependent variable and SPECIES, HABITAT and VEGETATION.

were the factors. All the calculations were performed using IBM SPSS Statistics for Windows (IBM Corp. Released, 2020). Throughout the text, mean values are presented with 95% confidence limits (CL).

### Phylogeny reconstruction

To visualise the species affiliation of the sequences obtained in the present study, phylogenetic reconstruction was carried out using the Bayesian approach. In MrBayes 3.2.7 (Ronquist et al. 2012) analysis was performed with 10 000 000 MCMC steps with 25% burn-in, two independent runs (each with four chains) starting from random trees were applied and trees were sampled every 100th generation. GTR + G + I substitution model was used as the best-fit model selected with jModelTest 2.1.10 (Darriba et al. 2012). Analyses finished when the average standard deviation of split frequencies was stabilised below 0.01 for all trees used to construct the final consensus tree. Finally, a phylogenetic tree was visualised using Figtree v. 1.4.4 (Rambaut et al. 2016).

## Results

### Collection of ticks

A total of 161 ticks including 115 *I. ricinus* (34 males, 51 females, 30 nymphs) and 46 *D. reticulatus* (20 males, 26 females) were collected (Table 1). Ticks were present at 29 (40.3% CL: 28.9–52.5) out of the 72 study plots. The average density of ticks/100m^2^ was 1.6 in parks, 0.5 in schools and kindergartens, 0.8 in urban greenery and 1.7 in forests.

**Table 1:** Total number of ticks detected in Zielona Góra in 2021 regarding to the habitat and the height of vegetation.

Tick species	Tick stage	I. Parks	II. Schools and kindergartens	III. Squares	IV. Forests	low	medium	high
*I. ricinus*	**male** (34, 21.1%)	12	2	18	2	9	1	24
	**female** (51, 31.7%)	19	8	14	10	15		36
	**nymph** (30, 18.6%)	21	2	3	4	3		27
*D. reticulatus*	**male** (20, 12.4%)	7	2	4	7	5	1	14
	**female** (26, 16.1%)	12	1	2	11	1	1	24
	Total	71	15	41	34	33	3	125
	[%]	44,1	9,3	25,5	21,1	20,5	1,9	77,6

**Table 2:** Total number of pathogens detected in Zielona Góra in 2021

Species	Stage	*B. afzelii*	*B. burgdorferi s.s*	*B. lusitaniae*	*B. miyamotoi*	*B. spielmanii*	*B. valaisiana*
*I. ricinus*	**male** (34, 21.1%)	4		2		2	
	**female** (51, 31.7%)	4	2	13	1	1	3
	**nymph** (30, 18.6%)	1		2		1	
*D. reticulatus*	**male** (20, 12.4%)	1		2			
	**female** (26, 16.1%)	1		2			
Total		11	2	21	1	4	3

**Fig. 2:**
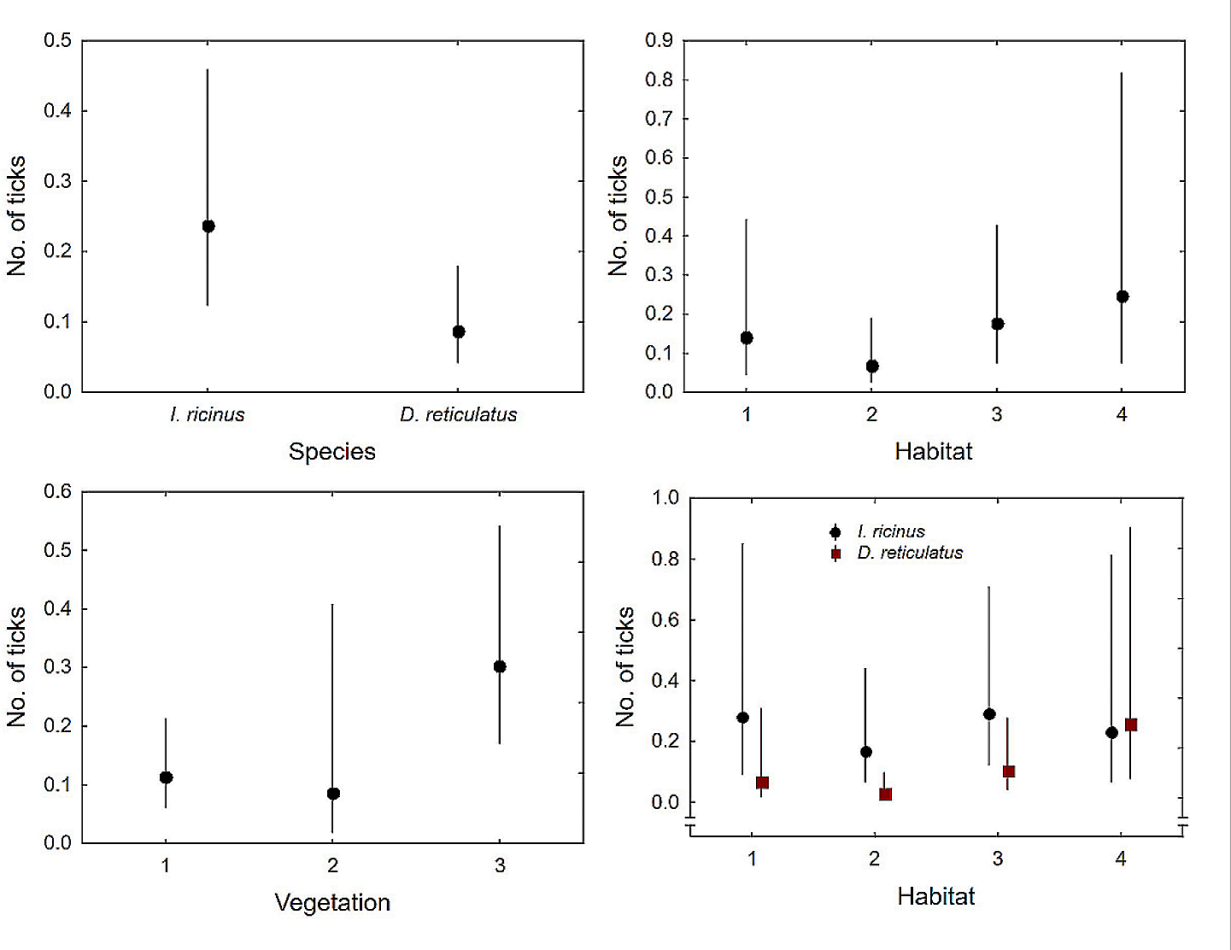
Number of ticks concerning tick species (**a**) habitat (**b**; 1– schools and kindergartens, 2– squares, 3– parks, 4– forests), height of vegetation (**c**; 1– low, up to 0.2 m, 2– medium: 0.21–0.4 m, 3– high: above 0.41 m) and interaction of species and habitat (d); mean values are presented with 95% confidence limits

The mean number of ticks per sampled plot (area of 100 m^2^) and sampling day was 1.12 (CL: 0.61–1.62, *n* = 144) and varied between 0 and 21. We found significant differences in the occurrence between the tick species, with *I. ricinus* recorded in higher numbers than *D. reticulatus* (Table 3; Fig. 2a). Furthermore, no effect of HABITAT was found (Table 3; Fig. 2b), but the number of ticks was influenced by VEGETATION (Table 3; Fig. 2c). The interaction effect of SPECIES and HABITAT was also significant Table 3; Fig. 2d.).

**Table 3:** Summary of Generalized linear mixed model (GLMM) analysis for potential factors affecting the occurrence of ticks in study sites; covariation of random effects: ID– 1.76, Z = 3.10, *p* = 0.002; TIME– 0.53, Z = 1.81, *p* = 0.070

Factor	F	DF	*P*
SPECIES	20.34	1, 278	< 0.001
HABITAT	1.26	3, 278	0.288
VEGETATION	4.00	2, 278	0.019
SPECIES*HABITAT	4.41	2, 278	0.005

### *Borrelia* infection

**Fig. 3:**
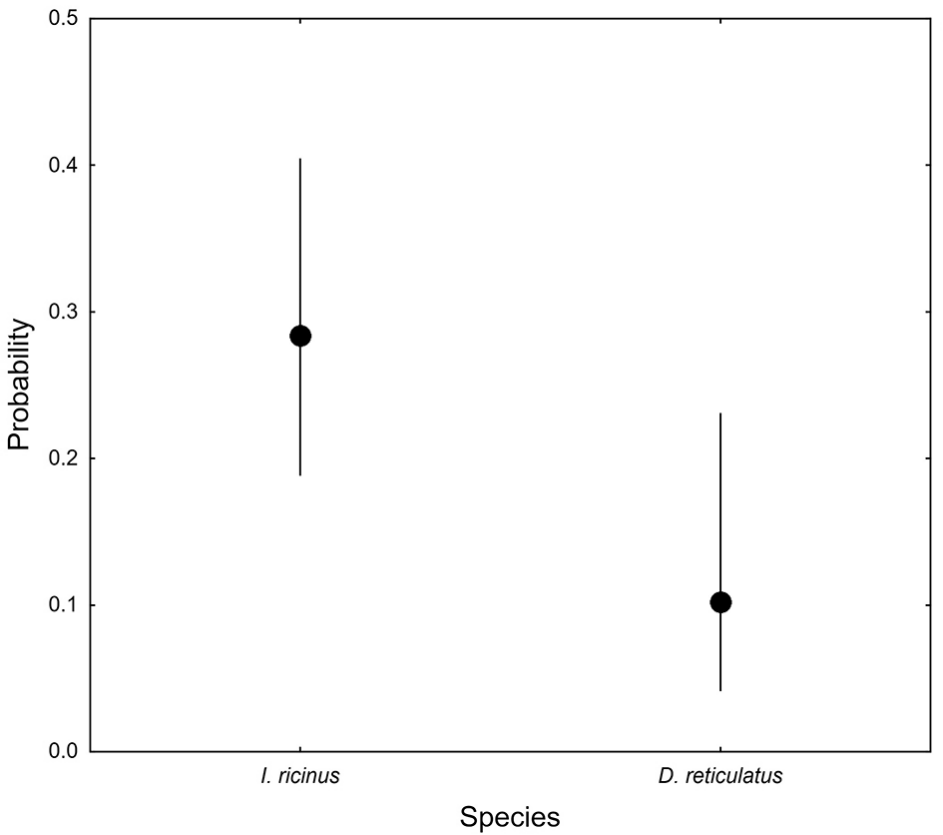
*Borrelia* spp. in a tick species; mean values are presented with 95% confidence limits

In total, *Borrelia* DNA was identified in 42 (26.1%) of the 161 ticks (CL: 0.195–0.336, *n* = 161), but the rate depended on the species (GLM, Wald chi-square = 6.09, df = 1, *p* = 0.014), with a much higher value in *I. ricinus* (Fig. 3). Furthermore, the occurrence of *Borrelia* spp. was not related to HABITAT, (GLM, Wald chi-square = 1.45, df = 3, *p* = 0.694) and VEGETATION (GLM, Wald chi-square = 1.45, df = 2, *p* = 0.982). 36 (85.7%) *I. ricinus* and six (14.3%) *D. reticulatus* yielded the bacterium. The presence of *B. burgdorferi* s.l. was detected at 16 of all study plots (22.2%). Of the PCR-positive samples successfully typed, 21 (50.0%) were *B. lusitaniae*, eleven (26.2%) *B. afzelii*, four (9.5%) *B. spielmanii*, three (7.1%) *B. valaisiana*, and two (4.8%) *B. burgdorferi* s.s. In only one study site the presence of *B. miyamotoi* was detected (1.4%). Of all PCR positive samples, *B. miyamotoi* accounted for 2.4% (Table 2). No co-infections were found. The dominant pathogen species was *B. lusitaniae* (51.2% cases in *B.burgdorferi* s.l. complex), *B. afzelii* (26.8%), *B. spielmanii* (9.8%), *B. valaisiana* (7.3%), and *B. burgdorferi* s.s. (4.9%).

**Fig. 4:**
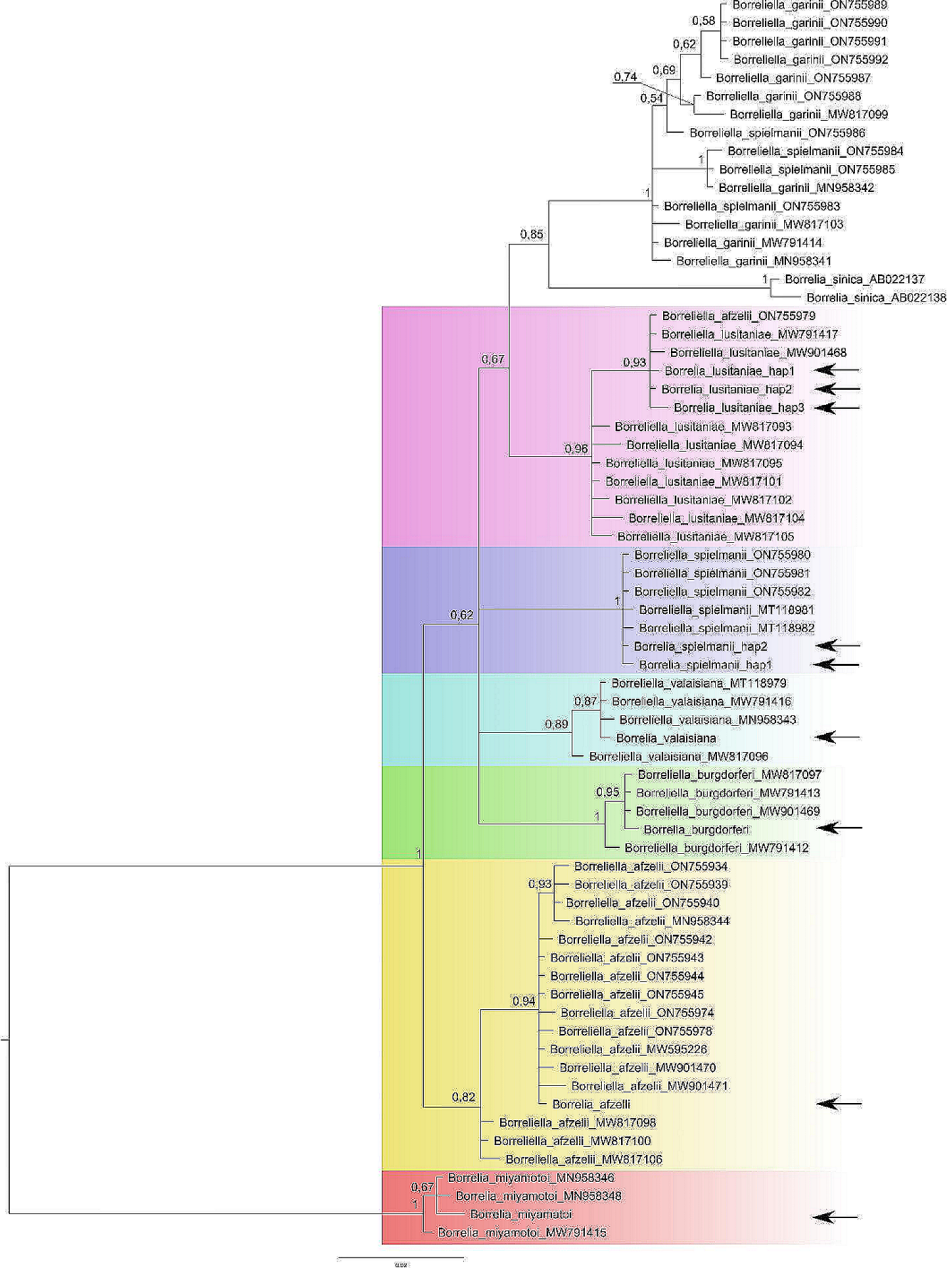
Bayesian phylogenetic tree presenting the systematic position of analysed DNA samples (indicated with an arrow). Numbers along the node are posterior probability values for nodes’ significance; sequences possessed from NCBI have accession numbers along with systematic names

Among all sequenced flagellin genes, we found one haplotype for *B. afzelii*, *B. burgdorferi* s.s., *B. valaisiana* and *B. miyamotoi*, two haplotypes for *B. spielmanii* and three for *B. lusitaniae.* All the haplotypes’ locations on the phylogenetic tree confirmed its systematic identification (Fig. 4). Furthermore, the highest number of spotted *Borrelia* species diversity was found in the study plots 49 and 38, with five different species each (Fig. 1).

## Discussion

The aim of our study was to determine the occurrence of ticks in urban green areas, with a focus on areas used by humans such as parks, schools and kindergartens, squares, and urban forests, and to assess the proportion of ticks infected with *Borrelia* spp., which is a very important issue in public health care. Zielona Góra which has quadrupled its area in recent years can be a model city to study the influence of urbanization on the distribution of ticks due to the way urban greenery is maintained and access to hosts such as small mammals and birds.

Our study indicates populations of two tick species in urban areas, both in the inner city and the city’s outskirts. In Zielona Góra, the two species *I. ricinus* and *D. reticulatus* were found using the flagging method. Ticks readily use urban greenery, and the number of species depends on latitude and other ecological factors. In a study conducted in Berlin (Rubel et al. 2022), as many as twelve tick species were found, and five species in Kraków (in this case ticks collected from the vegetation considered only two species– *I. ricinus* and *D. reticulatus*) (Kocoń et al. 2022). In Zielona Góra, the average density of all species of ticks was 1.1 individuals per 100 m^2^ (3.9 individuals per 100 m^2^ in positive squares); ticks were found in 43 out of 72 (40.3%) study sites. The average tick density appears to be low compared to data from other cities: 2–12 Katowice metropolitan area (Pet’ko et al. 1997), 8.9–11.1 Zabrze, Chorzów, Żywiec (Strzelczyk et al. 2015), Lublin 11.2 (Biaduń and Chybowski 2007), London 7.4 (Hansford et al. 2021), Warsaw 10.1 (Kowalec et al. 2017). But like in Olsztyn, the overall mean abundance was ca. 2.0 ticks per 100 m^2^ (Kubiak et al. 2019). The highest tick densities occurred in urban forests and city parks, serving as ecological corridors in the urban area. Urban greens (squares) and schools and kindergartens had lower tick densities, which may also be related to the lack of convenient ecological connections to the outer areas. Green corridors and inner-city parks are crucial for the persistence of tick populations within the city. It is from parks and green corridors that ticks, together with their hosts, enter smaller fragmented habitats such as school playing fields, especially when such corridors are well shaded and highly covered by vegetation. Urban green planning, therefore, should consider that ticks prefer damp and shaded areas, increasing the possibility of contact with people and hence the transmission of *Borrelia* spirochetes.

One of the objectives of the present study was to determine which habitat types of urbanized areas may be suitable for ticks infected with *Borrelia* spp., and which *Borrelia* species occur in the study area. The results showed that ticks infected with *Borrelia* spp. occur in 26.1% of the sample. plots Compared to other cities, the proportion is high: Kiev 4% (Didyk et al. 2017), Warsaw 10.9% (Kowalec et al. 2017), Luxembourg 11.3% (Reye et al. 2010), Salisbury 18.1% (Hansford et al. 2017), Olsztyn 27.1% (Kubiak et al. 2019), Hanover 22.7% (Tappe et al. 2014), and Hamburg (34.1%) (May et al. 2015). The presence of *Borrelia* spp. spirochetes of human epidemiological significance were found at 13.9% of all plots within the city. All of these were located on the city’s outskirts or were connections to the outskirts from green areas and parks on the west side of Zielona Góra. This may suggest that infected ticks are transmitted into these areas by free-living animals, especially small mammals of the micromammalia group as well as boar, deer and birds. Among birds, it is important to distinguish between ground feeders and those foraging in low vegetation (Ciebiera et al. 2019). The absence of *Borrelia* spp. in green spaces located next to playgrounds and kindergartens in the city center indicates that this pathogen did not expanded fully to the city core yet. This aspect needs further investigation. Nevertheless, an important point is an adequate management of urban green spaces to suppress tick populations (Stafford III 2007). This information can also be used in tick awareness campaigns among city residents by people with relevant knowledge and experience of tick populations.

The most common pathogen during our study was spirochetes of the *B. burgdorferi* s.l. In the *B. burgdorferi* s.l. group, our study confirmed the presence of the species *B. lusitaniae* (51.2% of cases in this group), *B. afzelii* (26.8%), *B. spielmanii* (9.8%), *B. valaisiana* (7.3%), *B. burgdorferi* s.s. (4.9%). In Poland, the *I. ricinus* infection rate of *B. burgdorferi* s.l. varies from 1.2 to 27.4% depending on the region (Karbowiak et al. 2018; Kubiak et al. 2019) and in Europe (3.6–19.3%) (Strnad et al. 2017). The high proportion of *B. lusitaniae* indicates a significant expansion of lizards in the city. The results of this study confirm the dominance of *B. lusitaniae* in ticks taken from the urbanised area of Zielona Góra. The occurrence of this species is confirmed in central Europe. However, southern Europe and the Mediterranean basin, including Portugal, Morocco, Tunisia, and Turkey, are the areas where *B. lusitaniae* is the dominant genospecies (Wang 2015). *B. lusitaniae* was rarely found in the continental Europe (Wodecka and Skotarczak 2015). The high prevalence of *B. lusitaniae* might be associated with environmental conditions, such as the region’s changing climate. In contrast, rodents are the main reservoirs of *B. afzelii, B. spielmanii* and birds are the main reservoir of *B. valaisiana* (Wang 2015). The different species of the pathogen in Zielona Góra show a tendency to flow into a strictly urbanised area.

*B. lusitaniae* is a species that is increasingly detected in Europe, not only in the countries of the Mediterranean basin. It is also found in Slovakia, Moldova, Bulgaria, and Ukraine (Postic et al. 1997; Gern et al. 1999; Christova et al. 2001; Weiner et al. 2018). Studies in Slovakia as well as in Serbia showed that *B. lusitaniae* was the predominant species in these areas (Taragel’ova et al. 2016, Cakic´ et al. 2019). The latest study by Cirkovic et al. (2024) has confirmed the increasing tendency of this species to spread over the last 15 years. One of the reasons may be the observed increase in the number of hosts of this species (Norte et al. 2015; Sukara et al. 2018), as well as global warming, which affects the distribution of this species (Moreno-Rueda et al. 2012). Presumably, this phenomenon is observed in western Poland. Our current investigation in Zielona Góra indicates that *B. lusitaniae* dominates in this area, followed by *B. afzelii*.

Surprisingly, we did not observe the occurrence of *B. garinii* in the study area, which is an interesting finding compared to other studies in central Europe. Individual scientific studies show different proportions of *B. garinii* in ticks found in recreational, urbanised or seminatural areas. *B. garinii* did not occurred in a green urban area of Olsztyn (north-eastern Poland) (Kubiak 2019). Data from Woliński National Park (north‑western Poland) shows that only 0.25% of 394 collected ticks were infected with *B. garinii* (Asman et al. 2021). Two infected ticks with *B. garinii* were found in urban recreational areas of Helsinki (Junttila et al. 1999). Three isolates were found in ticks collected from recreational areas in Eastern Slovakia (Štěpánová-Tresová et al. 2000). On the other hand, data from urban and suburban areas of Bonn shows that 27.9% of 250 ticks were infected with *B. garinii* (Maetzel et al. 2005). The lack of the typically second common species of *Borrelia* may be caused by environmental changes that limit the occurrence of birds, e.g. green areas management. How green areas are managed, the short-cutting of grass, the introduction of alien plant species and the availability of food for birds in rubbish bins are (among others) causing a homogenisation of bird biodiversity in the urban environment and have the highest influence on birds’ foraging behavior. This is perhaps the primary factor in the absence of *B. garinii* within the city, which birds are the main reservoir.

Males and females of *D. reticulatus* were responsible for the transmission of two pathogens: *B. afzelii* and *B. lusitaniae*. It is most likely that the larvae and nymphs of this species feeding on small mammals and lizards become infected with the spirochetes and transstadially transmit the pathogens to the adult stages, which in turn feed on large vertebrates (deer, wild boar, foxes) constantly present within the city (Hoornstra et al. 2022b).

On the other hand, the rarest *Borrelia* spp. representative, with only one record in Zielona Góra, was *B. miyamotoi*. This proportion is similar to other studies in Europe (Hansford et al. 2017). *B. miyamotoi* in contrast to *B. burgdorferi* s.l complex is classified into different phylogenetic groups, to the relapsing fever-like spirochetes. This bacterium is a new emerging tick-borne pathogen in the Northern Hemisphere responsible for *B. miyamotoi* disease (BMD) (Cleveland et al. 2023).

## Conclusions

The study focuses on the city of Zielona Góra as a model city. It indicates the presence of a well-established tick population in both the inner city and the outskirts. Two species of ticks, *I. ricinus* and *D. reticulatus*, were found. The average density of ticks in Zielona Góra was relatively low compared to other cities. Still, infection rate with *Borrelia* spp. was relatively high in the study area. The investigation also aimed to determine which habitat types in urban areas are suited for ticks infected with *Borrelia* spp. Infected ticks were present in 26.1% of all samples, with the highest prevalence in areas located on the outskirts of the city or those with ecological connections to the outskirts. Free-living animals, including small mammals, boar, deer, and birds, likely facilitated the introduction of infected ticks into our study area. *B. burgodrferi* s.l. group was the most common pathogen, with *B.lusitaniae* being the dominant species in the tick population. We stress the need for proper management of urban green spaces to suppress tick populations and raise awareness among city residents about ticks and tick-borne diseases.

## Data Availability

The datasets generated during and/or analysed during the current study are available from the corresponding author on reasonable request.
